# Thoracic radiography of healthy captive male and female Squirrel monkey (*Saimiri* spp.)

**DOI:** 10.1371/journal.pone.0201646

**Published:** 2018-08-07

**Authors:** Blandine Houdellier, Véronique Liekens, Pascale Smets, Tim Bouts, Jimmy H. Saunders

**Affiliations:** 1 Department of Medical Imaging and Small Animal Orthopaedic, Faculty of Veterinary Medicine, Gent University, Merelbeke, Belgium; 2 Department of Small Animal Medicine, Faculty of Veterinary Medicine, Gent University, Merelbeke, Belgium; 3 Zoo of Pairi Daiza, Brugelette, Belgium; University of Pretoria, SOUTH AFRICA

## Abstract

The purpose of this prospective study was to describe the normal anatomy and provide reference ranges for measurements of thoracic radiography on Squirrel monkeys (n = 13). Thoracic radiography is a common non-invasive diagnostic tool for both cardiac and non-cardiac thoracic structures. Furthermore cardiac disease is a common condition in captive primates. In this study, left-right lateral, right-left lateral and dorsoventral projections of 13 healthy Squirrel monkeys were reviewed during their annual health examinations. The mean Vertebral Heart Score on the left-right and right-left lateral projections were 8,98 ± 0,25 and 8,85 ± 0,35 respectively. The cardio-thoracic ratio on the dorsoventral projection was 0,68 ± 0,03. The trachea to inlet ratio was 0,33 ± 0,04. Other measurements are provided for the skeletal, cardiac and respiratory systems. Knowledge of the normal radiographic thoracic anatomy is fundamental in clinical as well as research settings for accurate diagnosis of diseases.

## Introduction

Squirrel monkeys are arboreal neotropical monkeys, belonging to the family of Cebidae and subfamily Saimirinae. Many genetic sequence data have been obtained from the currently organized squirrel monkey taxa: *S*. *oerstedii oerstedii*, *S*. *o*. *citronellus*, *S*. *cassiquiarensis cassiquiarensis*, *S*. *c*. *albigena*, *S*. *macrodon*, *S*. *ustus*, *S*. *sciureus sciureus*, *S*. *s*. *collinsi*, *S*. *boliviensis boliviensis*, *S*. *b*. *peruviensis*, and *S*. *vanzolinii* [1].

Squirrel monkeys are useful animal models in a variety of different disciplines in biomedical research due to their phylogenetic similarities to humans [2]. Many studies have been published in order to better understand the basic biology [2–5], biogeography [3, 6], morphology [2, 3, 5–8] and also behavior [2, 9, 10] of this species. However, research has still to be done to better understand the biology and morphology of this species.

Cardiovascular diseases are particularly overrepresented in monkeys, having been identified as a major cause of death in squirrel monkeys particularly [11], in other primates [12] and captive great apes [13]. Reviews of mortality report that cardiovascular diseases represent 41% of deaths in gorillas [14], and 81,25% in aged chimpanzee [15]. Cases of dilated (DCM) and hypertrophic cardiomyopathy (HCM) like phenotype have been reported in aged Squirrel monkeys [11, 16]. High prevalence of DCM has also been demonstrated in other small monkeys [12, 17]. Multiple reviews of fibrosing cardiomyopathy are reported in multiple species [17–21] and a strong genetic predisposition in Rhesus macaques (*Macaca Mulatta*) for HCM has been demonstrated [22].

Because of the importance of cardiac disease in this species, previous research concentrated on electrocardiographic and echocardiographic characteristics of Squirrel monkeys [23]. Some publications are also available on smaller monkeys like Francois Langurs (*Trachypithecus francoisi*) [17]. Echocardiography may be the preferred examination for cardiac disease, but thoracic radiography is a quicker and easier examination that can give a lot of basic information on both cardiac and non-cardiac thoracic structures. Thoracic radiography is essential to access lower airway disease and is commonly used in non-human primates. The radiographic thoracic anatomy has been studied in many other more or less similar species like ring-tailed lemur (*Lemur catta*) [24], Goeldi’s monkey (*Callimico goeldi*) [25], Rhesus macaque [26], Cynomolgus monkey (*Macaca fascicularis*) [27], pet macaques of Sulawesi *(Macaca nigra* and *tonkeana*)[28], vervet monkey (*chlorocebus sabaeus*) [29] and also in close species as in capuchin (*Cebus paella*) [30]. The tracheal morphology has also been studied in *Saimiri* spp. [31]. Lower airway disease has been reported as viral and bacterial infection in chimpanzees and macaques [32]. Toxoplasmosis has been reported in squirrel monkeys [33, 34] and bronchopneumonia has been demonstrated to be the most frequent cause of death in older animals [35].

The aim of this study is to describe the normal radiographic thoracic anatomy of the *Saimiri* spp. as a species-specific reference.

## Materials and methods

All examinations were performed on site in the veterinary hospital (Pairi Daiza, Brugelette, Belgium). The data used in the study were collected during the annual health examination, that includes physical examination, blood sampling, radiography of the thorax and echocardiography, of the animals.

### Animals

Fourteen captive Squirrel monkeys (*Saimiri* spp.), 9 *Saimiri boliviensis peruviensis* and 5 *Saimiri scireus*, from Pairi Daiza, Belgium were submitted for a full clinical examination including cardiac biomarkers (N Terminal-prohormone of Brain Natriuritic peptide “NT-proBNP” and troponin I, tests performed by Electrochemiluminescence “ECLIA”) and complete echocardiographic examination performed by a board-certified cardiologist for evaluation of the cardiovascular system (PS). One *Saimiri boliviensis peruviensis* presented a significant abnormality (severe dilation of the ascending aorta) on echocardiography and was excluded from the study. The remaining 13 monkeys were included in the study.

The age of the animals ranged from 2,25 to 21,3 years (mean age = 9,12 years). The minimum and maximum weights of the animals were 467 g and 756 g respectively (mean weight = 615 g). There were 11 females and 2 males. All 14 animals are housed in a combined indoor (200 square meters) and outdoor (islands of 1360 and 1650 square metres) enclosure with multiple environmental enrichments such as ropes and sticks. The environment is natural with trees and plants, and their food is based on fruits, vegetables and primates diet (MAZURI®).

This study adhered to the legal requirements of Belgium and is in accordance with the recommendations of the Weatherall report on the use of non-human primates.

### Anaesthesia

No animals were anesthetized for any other reasons than their general health examinations.

Examinations were performed under general anaesthesia. Induction and maintenance of anaesthesia was by mask. An Ayre’s T-piece was used for all animals to minimize resistance and dead space. The volume of oxygen was kept at 2 L/min at all times and the percentage of isoflurane (Isoflo, Ecuphar) varied depending on the anaesthetic monitoring of the patient. The percentage was on the maximum value (5%) for induction and was slowly decreased until 1,5–2% for maintenance. During the entire anaesthesia, heart rate and oxygen saturation was monitored by pulse-oxymetry placed on the hand, respiratory rate was counted manually and rectal temperature was continuously monitored with a digital probe. Anaesthetic depth was monitored by checking eyelid (medial palpebral reflex) and cornea reflexes. Individual anaesthetic files were kept for all animals. Due to the small size of the animals, maintaining the temperature was the main challenge. Plastic gloves filled with hot water (37°C), a heating carpet (38°C) as well as pre-heated sonographic gel were used. All animals received 20 mL of saline solution (Ringer Lactate^®^ solution) subcutaneously at the beginning of anaesthesia. After anaesthesia, animals were allowed to recover in a small dark transport cage in a heated room before being transported back to their enclosure. Animals were monitored after anaesthesia for 3 days without adverse effects.

After radiographic examinations, all animals underwent a complete echocardiographic examination by a board-certified cardiologist and an electrocardiogram (ECG) with the Alivecor system (Veterinary iPhone® ECG and heart monitor). The minimum and maximum time of anesthesia were 22 minutes and 35 minutes respectively (mean time = 31 minutes).

### Radiography

Left-right lateral (RL), right-left lateral (LL) and Dorsoventral (DV) radiographic projections of the thorax were taken at the end of inspiration using a direct digital radiography device (Sound-Eklin®). On one animal (*Saimiri boliviensis peruviensis*), the head was superimposed with the cranial aspect of the thoracic cavity on the DV projection due to the short neck conformation. On one patient, a straight DV projection could not be performed. The Source to Image Distance (SID) was settled at 100 cm and images were obtained with 60 kVp and 2,5 mAs. The radiographs were stored in DICOM format and were archived to a PACS system.

### Criteria for interpretation

All the radiographic images were analyzed by the same observer (BH), that evaluated all the thoracic structures individually.

**Fig 1:**
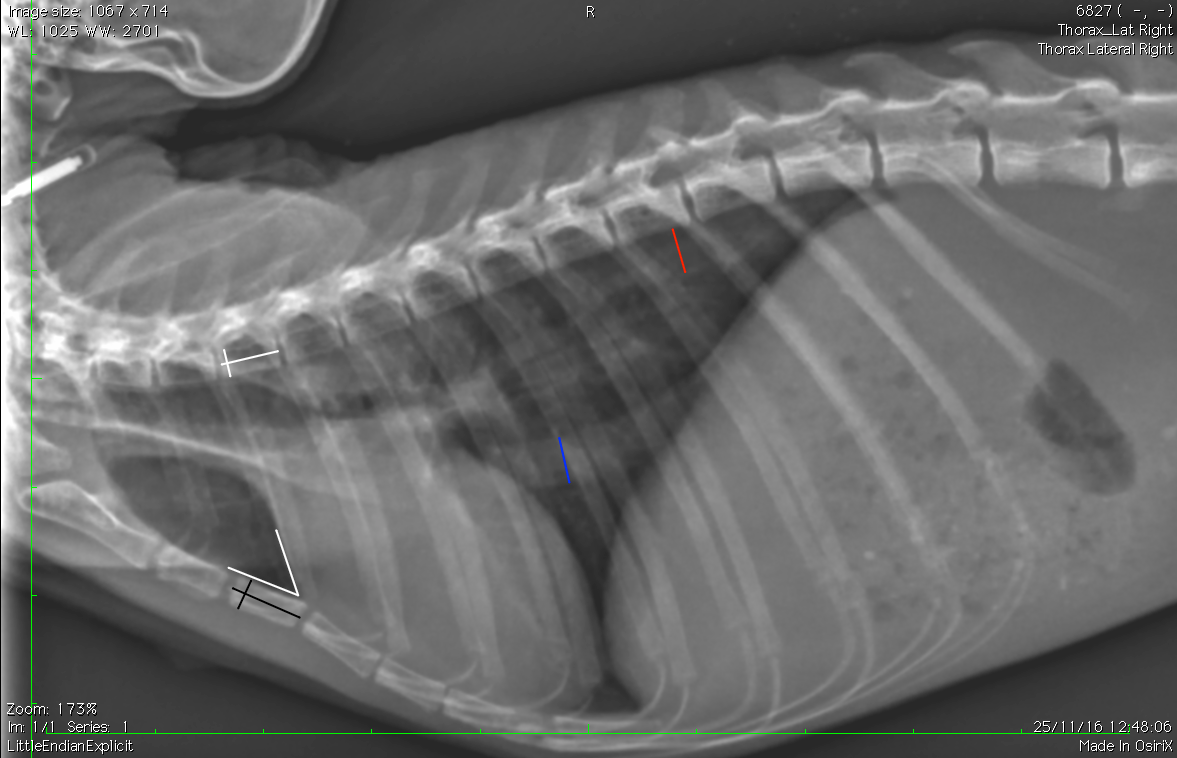
Left-right lateral projection of a 9 years old *Saimiri sciureus* showing the measurements of the length and height of the thoracic vertebrae (white lines) and sternebrae (black lines). The diameter of the aorta (red line) and caudal vena cava (blue line) are also presented. The angle of cardiac inclination is represented by the two white lines between the cranial border of the cardiac silhouette and the sternebrae.

**Fig 2:**
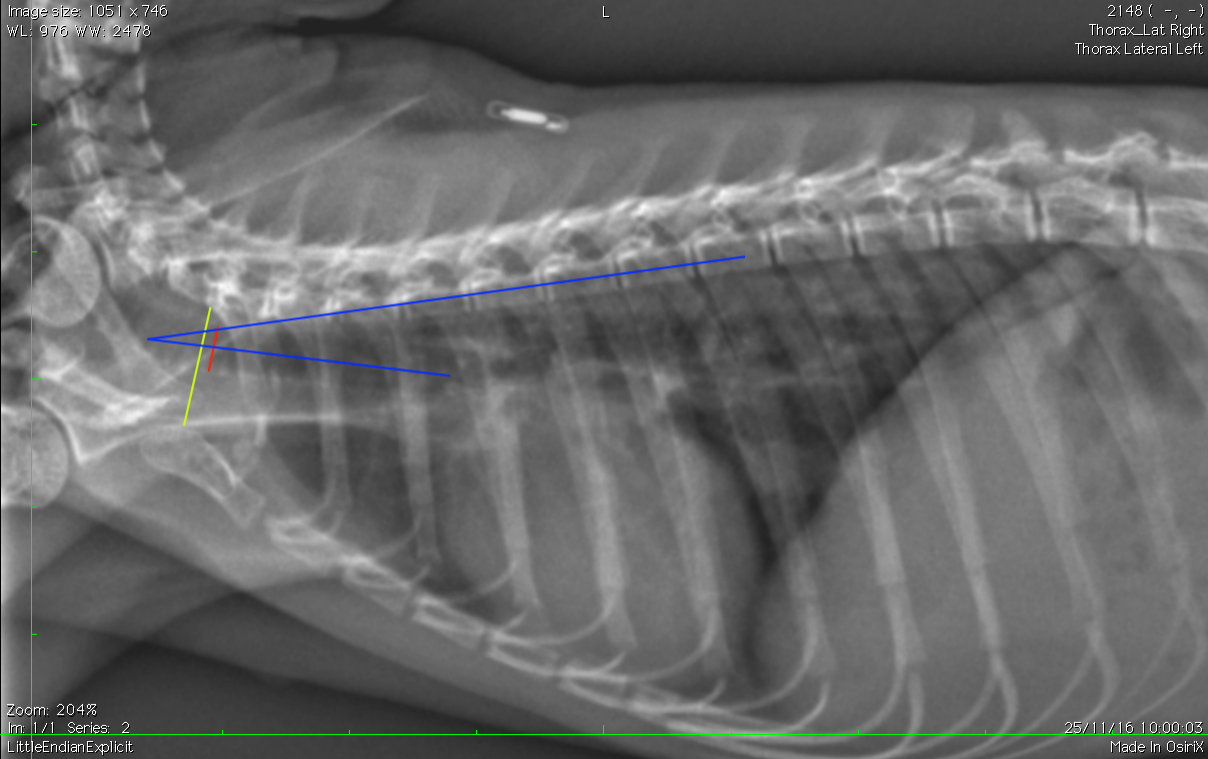
Right-left lateral projection of a 4 year old *Saimiri boliviensis peruviensis*. Measurements of tracheal diameter (red line) to thoracic inlet length (yellow line) ratio; tracheal inclination (blue lines) are presented. Note the carina located at the level of the third intercostal space.

**Fig 3:**
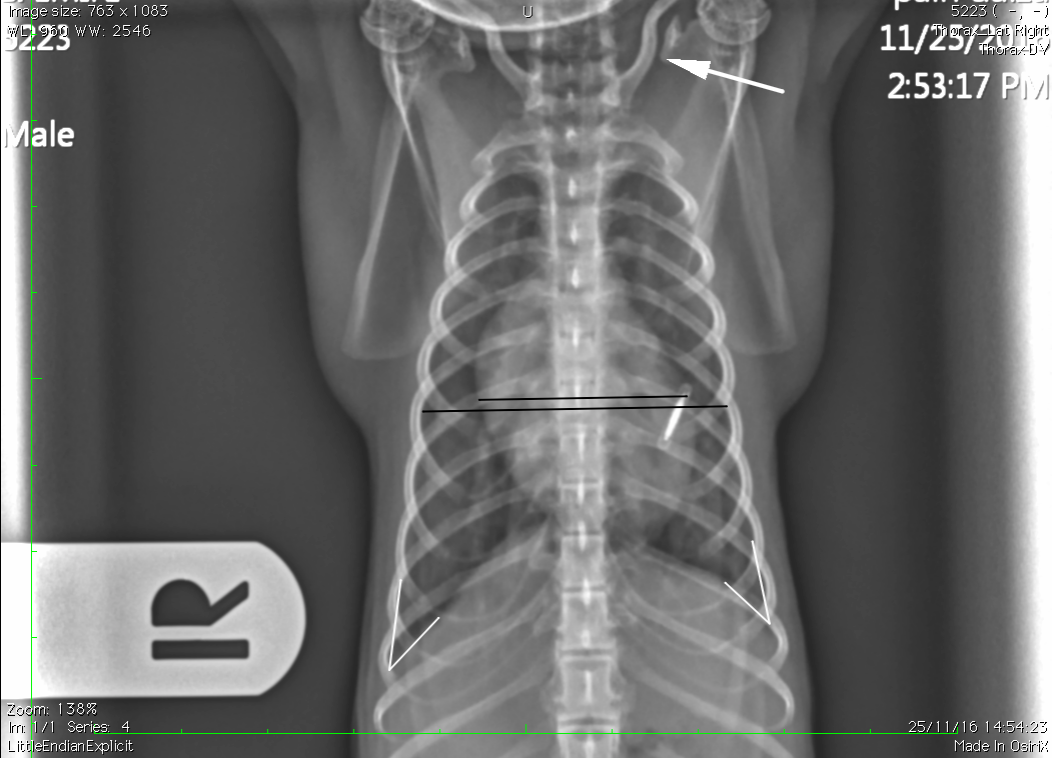
Dorsoventral projection of a 2 year old *Saimiri boliviensis peruviensis*. The cardio-thoracic ratio is shown with the black lines; The left and right costophrenic angles are visualized with the white lines where the diaphragm meets the ribs. Note the presence of a clavicula (white arrow).

**Fig 4:**
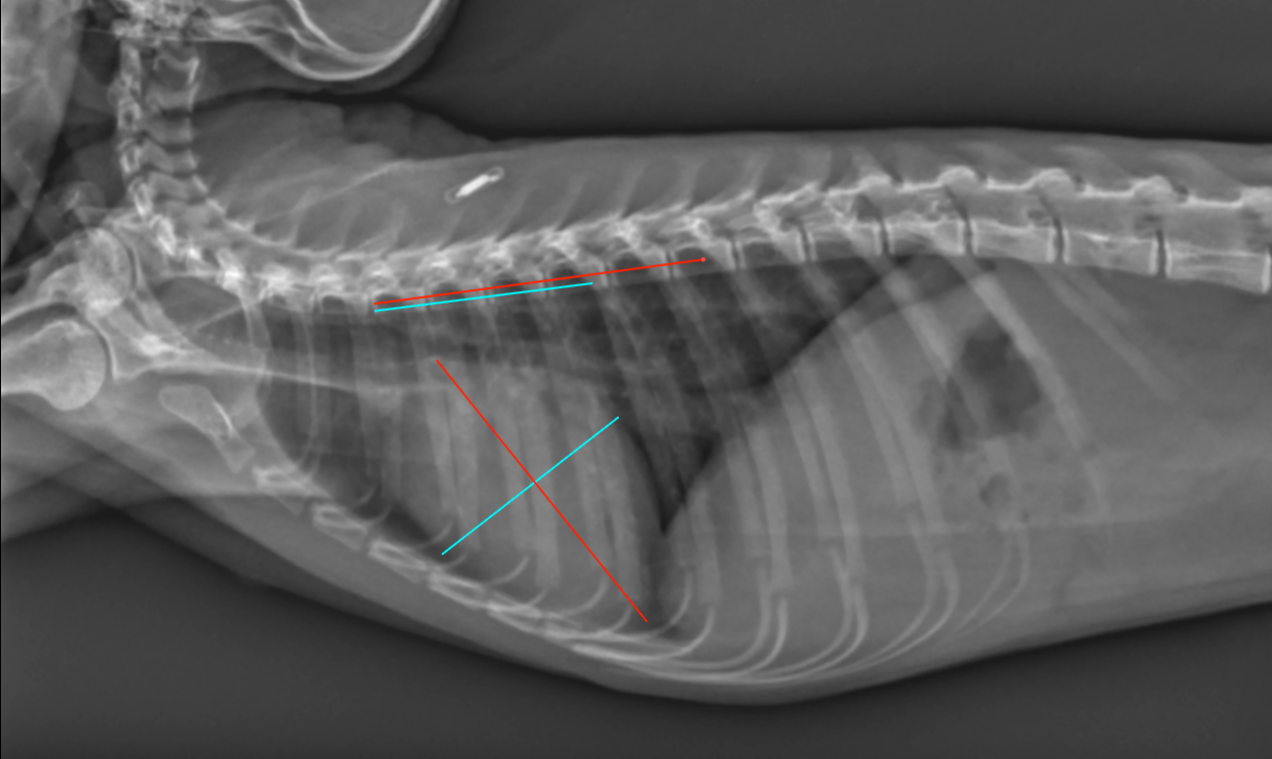
Left-right lateral thoracic radiograph of a 4y old female *Saimiri boliviensis peruviensis* illustrating the measurement of the VHS (Vertebral Heart Score).

- The presence or absence of a clavicula was recorded.
- The number of ribs (including floating ribs), vertebrae (including the anticlinal vertebra) and sternebrae were recorded. The length and height of the vertebrae and sternebrae were measured on the RL projection from the midpoint of the cranial end plate to the caudal end plate (length) (Fig 1) and along the cranial endplates (height) [24].
- The tracheal diameter and more precisely the trachea to thoracic inlet ratio was calculated as proposed in dogs and monkeys [24, 36]. The tracheal inclination was calculated as the angle formed by the ventral surface of the bodies of the thoracic vertebrae and the trachea [30]. The location of the carina was also defined (Fig 2).
- For evaluation of the cardiac size, the ratio of the cardiac height/thoracic height on the lateral projections, as well as the width on the DV projection (at the widest point of the cardiac silhouette) have been calculated (Fig 3). The number of intercostal space occupied by the cardiac silhouette has also been reported in order to have multiple measurements available and to be more accurate.
- The angle of cardiac inclination is formed by the right cardiac border and the sternum on the lateral projection as described [24, 30, 37] (Fig 1). The number of sternebrae in contact with the cardiac silhouette has also been calculated.
- The Vertebral Heart Score (VHS) was measured on the RL (RL-VHS) and LL (LL-VHS) projections according to the protocol established in dogs [38] (Fig 4).
- The costophrenic angles were measured on the right (RCA) and left side (LCA) on the DV projection and were defined where the diaphragm meets the ribs [30] (Fig 3).
- The major vessels diameter refers to aorta (AO) diameter and caudal vena cava (CVC) diameter on a lateral projection (Fig 1). The projection was chosen where the edges of the vessel were better visualized. We assumed that the diameter would not change significantly from one lateral to another giving the small size of the patient. The ratio CVC/AO was measured as published in dogs [39].
- The ratio between the width of the thoracic vertebrae and cranial mediastinum has been recorded on the DV projection (Fig 3).

## Results

### Relevant skeletal system

**Fig 5:**
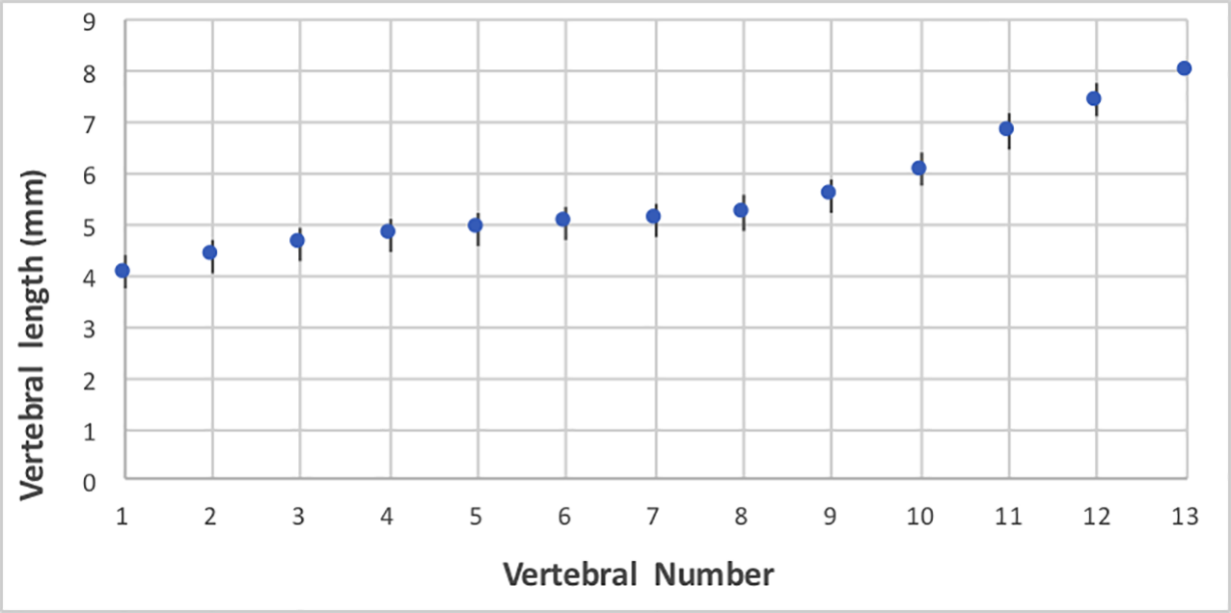
Means and standard deviations of radiographic vertebral body lengths in 13 *Saimiri* spp. Note the rapid increase in vertebral body length caudal to T9.

All the animal had 13 thoracic vertebrae with presence of 13 pair of ribs and two pairs (12/13 animals) or one pair (1/13) of floating ribs. One male demonstrated an elongated transverse process of L1 on the right side. The caudal thoracic vertebral bodies demonstrated larger length and width compared to the cranial thoracic vertebral bodies (Fig 5) as published in vervet monkeys [29]. The anticlinical vertebra was always T11 as in most dogs [40].

An average of 7,23 sternebrae (range: 6–8) were present in all the monkeys, including the manubrium and xyphoid. The average of the length and width of the third sternebrae were 5,72 mm ± 0,86 mm (range: 4,18–6,86 mm) and 2,2 mm ± 0,35 mm (range: 1,78–2,82 mm) respectively. The values were measured on the third sternebrae because it was better visualized on all the examinations.

The claviculae were present in all the 13 monkeys (Fig 3).

All the radiographic findings of the skeletal system are summarized in Table 1.

**Table 1:** Summarized radiographic measurements (mm) and findings of the skeletal system of 13 healthy anesthetized *Saimiri* spp.

	Variables	Mean ± SD	Range (min-max)
**T1**	Length	4,07 ± 0,36	3,6–4,86
	Height	2,08±0,23	1,46–2,43
**T2**	Length	4,39 ± 0,32	3,92–5,06
	Height	2,17±0,17	1,96–2,53
**T3**	Length	4,62 ± 0,37	3,91–5,11
	Height	2,30±0,23	2,05–2,73
**T4**	Length	4,79 ± 0,31	4,04–5,32
	Height	2,26±0,29	1,95–2,92
**T5**	Length	4,91 ± 0,34	4,22–5,47
	Height	2,26±0,29	1,95–2,92
**T6**	Length	5,02 ± 0,36	4,3–5,62
	Height	2,60±0,33	2,11–3,14
**T7**	Length	5,09 ± 0,36	4,3–5,59
	Height	2,77±0,35	2,18–3,34
**T8**	Length	5,23 ± 0,35	4,76–6,04
	Height	2,82±0,36	2,09–3,38
**T9**	Length	5,55 ± 0,30	4,53–5,32
	Height	3,02±0,29	2,65–3,44
**T10**	Length	6,06 ± 0,29	5,66–6,59
	Height	3,17±0,37	2,72–3,94
**T11**	Length	6,82 ± 0,36	6,33–7,48
	Height	3,28 ± 0,40	2,61–3,91
**T12**	Length	7,43 ± 0,38	6,75–8,22
	Height	3,31 ± 0,39	2,57–3,91
**T13**	Length	8,02 ± 0,22	7,36–9,06
	Height	3,54 ± 0,33	2,88–3,91
**Anticlinal vertebrae**		11	
**St 3**	Length	5,72±0,86	4,18–6,86
	Height	2,2±0,35	1,78–2,82
**Number of St**		7,23 ± 0,73	6–8
**Number of ribs**	Total	13	
	Floating	1–2	

T: Thoracic vertebrae. St: Sternebrae.

### Cardio-vascular system

#### Left-right lateral (RL) projection

The length of the long axis (height) and short axis (width) of the cardiac silhouette on the RL projection were 2,95 ± 0,23 cm (range: 2,54–3,32) and 2,05 ± 0,12 cm (range: 1,86–2,29) respectively. The mean RL-VHS was 8,98 ± 0,25 (range: 8,5–9,3). The cardio thoracic ratio was 0,76 ± 0,04 (range: 0,69–0,82). The number of intercostal spaces occupied by the cardiac silhouette was 3,08 ± 0,34 (range: 2,5–3,5 spaces). The number of sternebrae in contact with the cardiac silhouette was 3,08 ± 0,19 (range: 3–3,5 sternebrae). The angle of cardiac inclination was 35,43° ± 7,06° (range: 25,5–46,01).

#### Right-left lateral (LL) projection

The height and width of the cardiac silhouette were 2,81 ± 0,16 cm (range: 2,52–3,05) and 2,13 ± 0,18 cm (range: 1,8–2,34) respectively. The mean LL-VHS was 8,85 ± 0,35 (range: 8,6–9,5).

#### Dorsoventral (DV) projection

The apex of the heart was always positioned to the left. The width of the cardiac silhouette at the widest point was 2,38 ± 0,17 cm (range: 2,05–2,74) and the thoracic width taken at the same level was 3,49 ± 0,17 cm (range: 3,29–3,9). The cardio-thoracic ratio was then 0,68 ± 0,03 (range: 0,61–0,71).

The radiographic findings of the cardiac system are summarized in Table 2.

**Table 2:** Radiographic findings (cm) of the cardiac system of healthy anesthetized *Saimiri* spp.

	Projection	Variables	Mean ± SD	Range (min-max)
**Cardiac silhouette**	RL	Height	2,95 ± 0,23	2,54–3,32
		Width	2,05 ± 0,12	1,86–2,86
**VHS**			8,98 ± 0,25	8,5–9,3
**Thoracic size**		Height	3,89 ± 0,31	3,36–4,32
**Cardio-thoracic ratio**			0,76 ± 0,04	0,69–0,82
**Number of intercostal spaces**			3,08 ± 0,34	2,5–3,5
**Number of sternebrae in contact**			3,08 ± 0,19	3–3,5
**Angle of cardiac inclination**			35,43 ± 7,06	25,5–46,01
**Cardiac silhouette**	LL	Height	2,81 ± 0,16	2,52–3,05
		Width	2,13 ± 0,18	1,8–2,34
**VHS**			8,85 ± 0,35	8,6–9,5
**Cardiac size**	DV	Width	2,38 ± 0,17	2,05–2,74
**Thoracic size**		Width	3,49 ± 0,17	3,29–3,9
**Cardio-thoracic ratio**			0,68 ± 0,03	0,61–0,71

VHS: Vertebral Heart Score, RL: left-right Lateral, LL: right-left Lateral, DV: dorsoventral

The caudal vena cava was better visualized and delineated in right-left lateral projection in all patients. Compared to the caudal vena cava, the aorta was less conspicuous and the edges were less clear in all patients. The mean aortic diameter was 4,14 mm ± 0,36 mm and caudal vena cava was 4,47 mm ± 0,36 mm with the ratio CVC/AO at 1,08 ± 0,09 (range: 0,91–1,20) (Table 3). The pulmonary vessels were mildly visualized in 10/13 animals and barely visible in 3/13 animals. As for the major vessels, the pulmonary vessels were better visualized in right-left lateral projection.

**Table 3:** Radiographic anatomical measurements of the main vascular structures (mm).

	Mean ± SD	Range (min-max)
**Diameter AO**	4,14 ± 0,36	3,36–5,01
**Diameter CVC**	4,47 ± 0,36	3,90–4,82
**Ratio CVC/AO**	1,08 ± 0,09	0,91–1,20

### Respiratory system

Overall, a mild diffuse interstitial pattern was noted in 8/13 monkeys, independently of the body condition and the age. On the left-right lateral radiograph, the tip of the lung lobes ended at T11 (1/13), T12 (5/13), T13 (6/13) and L1 for 1/13 patients. On the DV projection the diaphragm ended at the level of T9 (8/13) or T10 (5/13) and the right and left costophrenic angles were respectively 33,01°± 5,29° and 34,40°± 7,11°.

In all the 13 monkeys, the tracheal rings were mineralized and the trachea was well visualized. The carina was seen at the level of the third intercostal space (10/13) or at the level of the fourth intercostal space (3/13). On the DV projection, the trachea was not consistently visualized and often superimposed with the vertebral bodies. The trachea to inlet ratio was 0,33 ± 0,04 (Table 4). The tracheal inclination was ranged between 10,04° and 19,7° with an average at 14,62° ± 3,44°. The radiographic findings for the respiratory system are summarized in the Table 4.

**Table 4:** Radiographic findings and measurements of the respiratory system. RCA: Right costophrenic angle, LCA: Left costophrenic angle.

	Mean ± SD	Range (min-max)
**RCA**	33,01 ± 5,29	27,39–42,94
**LCA**	34,40 ± 7,11	25,34–48,27
**Trachea/thoracic inlet ratio**	0,33 ± 0,04	0,24–0,37
**Tracheal inclination**	14,62 ± 3,44	10,04–19,7

## Discussion

A lot of characteristics make the squirrel monkey pertinent to research. Furthermore, many studies have been conducted in order improve the conservation of different subspecies of *Saimiri* spp. [41–43]. This study provides the first thoracic radiography data from anesthetized clinically healthy male and female squirrel monkeys and provides new references ranges. These indices are helpful in diagnosing respiratory and cardiovascular disorders and characterizing these disease processes in animal disease models [27]. The integrated readout system allows a faster image acquisition and images are directly sent to computer. In this case, the post processing of the images is very important due to the small size of the patient and non-optimal window and level. The DV projection was also chosen instead of VD because of the flat chest conformation of the patient; the DV was straighter and subjectively more repeatable. The neck was extended as much as possible on the DV projection.

Primates have different number of thoracic vertebrae depending on the species. The thoracic region is more variable compared to the cervical or lumbar, with the number of vertebrae varying between 11 to 14 [44]. In the present study, all the *Saimiri* spp. had 13 pairs of ribs, with 1 or 2 pairs of floating ribs. The gradual increase in caudal thoracic vertebrae has been reported previously in primates [29], and the rate of increase varies between species [45]. This difference could be significant in some measurements, as for the VHS. However, the vertebral length remained fairly constant between T4 and T9, where the normal VHS is measured. But the accuracy of the measurement should be studied in order to precise if this difference in vertebral length could affect the VHS in a monkey with cardiac disease for example.

A mild diffuse interstitial pattern was noted in a majority of *Saimiri* spp. This pattern is considered normal in some primates [29, 46]. Although all the projections were meant to be in inspiration, it was not always feasible due to the small size of the *Saimiri* spp. This factor could also partially explain the lung pattern and affects the location of the tip of the lungs. No signs of persisting atelectasis were otherwise reported as in other monkeys [29].

A positive pressure ventilation could not be applied because the animals were not intubated. Even if this is not ideal for the examination in inspiration, it had numerous advantages. Multiple drugs have been studied in primates demonstrating their effects on the cardiovascular system [47–49]. For example, in small primates, it was demonstrated that multiple anesthetic protocols resulted in increased blood pressure and heart rate, and the isoflurane protocol showed lower value of blood pressure compared to all other protocols [50]. Using only isoflurane allows to be closer to physiological circumstances and has been indicated for debilitated animals and prolonged procedures in squirrel monkey [2]. This finding is very important, especially for the cardiovascular system. In our case, the heart rate, respiratory rate and expired CO2 concentration were continuously monitored with pulse oxymetry present on the hand. The constant choice of the anesthetic protocol is important for the reproducibility of the results. The main problem encountered during anesthesia was hypothermia. Small body size, low body fat and long extremities contribute to rapid heat loss when these animals are anesthetized [2]. For this study, we used a heating pad, latex gloves filled with hot water and blankets. The temperature was also recorded during the entire anesthesia with a rectal probe connected directly to the anesthetic machine. The time of anesthesia was as short as possible, in order to decrease the anesthetic risk. The mean time for the three projections was 5 minutes with the total anesthetic time being 31 min ± 3,9 min (range: 22–35 min). The remaining time was used for the ECG recording and complete echocardiography. All the recoveries were smooth and without incidents.

The edges of the aorta were not always well visualized due to the superimposition of the pulmonary vessels and the interstitial pattern. However, a mean ratio with the diameter of the caudal vena cava could be calculated from the right or left lateral projection. In the present study, we assumed that the diameter would not change significantly from one lateral projection to another due to the small size of the patient. The projection was chosen in function of the good visualization of the edges. The ratio CVC/AO may be a useful tool for the diagnosis of right-sided cardiac failure in dogs [39]. The normal ratio can therefore give a reference value, but further studies have to be done in animals with right-sided heart disease. The pulmonary vessels were smaller and no definitive ratio with the ribs, as in dogs, could be presented.

The vertebral heart score was also calculated on the two lateral projections. In dogs, the RL-VHS is significantly higher compared to LL-VHS [51]. This is supposed to be a consequence of the magnification. In the present study, the RL-VHS was also mildly higher compared to LL-VHS but the small size made this difference insignificant. The other measurements were calculated from the RL projection because this is the most used lateral projection. The number of sternebrae in contact with the cardiac silhouette is also a useful tool as it can be increased in case of cardiomegaly [52]. Other measurements that could help in case of suspicion of cardiomegaly are for example dorsal deviation of the trachea with narrowing of the tracheal inclination [40].

The measurements are assumed to give reference ranges for healthy animals based on the following criteria: normal general behavior and physical examination, all the echocardiographic measurements were within available normal reference ranges as previously published[23], and normal cardiac troponin I values [53]. Cardiomyopathy and heart failure are the primarily health concern in the geriatric Saimiri spp. population[23], and more precisely HCM or DCM[11, 16]. The cardiac silhouette in these cases would appear enlarged, as a left sided or generalized cardiomegaly (increased VHS, elevation of the trachea, elongation of the left ventricle, rounding of the left heart border)[40]. Secondary signs of left-sided congestive cardiac failure (venous congestion and increased lung pattern) or right-sided congestive heart failure (hepatomegaly, CVC congestion and suspicion of ascites in the cranial abdomen included) can also be present.

### Limitations

There are several limitations in this study.

The small number of patients is the first limitation. There were a lot of variations in the age and weights. The low number of patients does not allow to assess statistical differences between different measurements, as for changes in cardiac size with age, cardiac inclination and shape. In cats, the cardiac size is proven to be larger in young cat, and more tortuous (tortuous aortic arch) with a pronounced cardiac inclination in aged cats for example [52]. No sex differences could be proven as well. Other studies have to be done with a larger group and a differentiation between male/female and also young/older animals.

In conclusion, knowing the normal measurements of thoracic radiographic parameters is essential for defining abnormalities. This study gives an overview of normal thoracic radiographic anatomy and gives primary reference values in *Saimiri* spp. monkeys. These findings are useful in veterinary medicine practice as well as in research involving non-human primate models of respiratory or cardiovascular disorders and morphologic studies on squirrel monkeys (*Saimiri* spp.).

## Supporting information

S1 Fig Left-right lateral projection of a 9 years old *Saimiri sciureus*. Size of the thoracic vertebrae, sternebrae, diameter of the aorta, caudal vena cava and angle of cardiac inclinication can be measured.
(TIF)

S2 Fig Right-left lateral projection of a 4 year old *Saimiri boliviensis peruviensis*. Tracheal diameter to thoracic inlet length ratio and tracheal inclination can be measured.
(TIF)

S3 Fig Dorsoventral projection of a 2 year old *Saimiri boliviensis peruviensis*. The cardio-thoracic, left and right costophrenic angles are measured. Note the presence of a clavicula.
(TIF)

S4 Fig Left-right lateral thoracic radiograph of a 4y old female *Saimiri boliviensis peruviensis*. Measurement of the VHS (Vertebral Heart Score).
(TIF)

S1 Supporting Information *Saimiri* spp. xlsx. Informations concerning the animals.
(XLSX)

S2 Supporting Information Raw data *Saimiri* spp. Xray.xlsx. Raw data of all 13 animals.
(XLSX)
