# Supplementary figures and images for: Thoracic radiography of healthy captive male and female Squirrel monkey (Saimiri spp.)
Source: PLoS One. 2018 Aug 7;13(8):e0201646. doi: 10.1371/journal.pone.0201646 (PMC6080787; doi:10.1371/journal.pone.0201646)

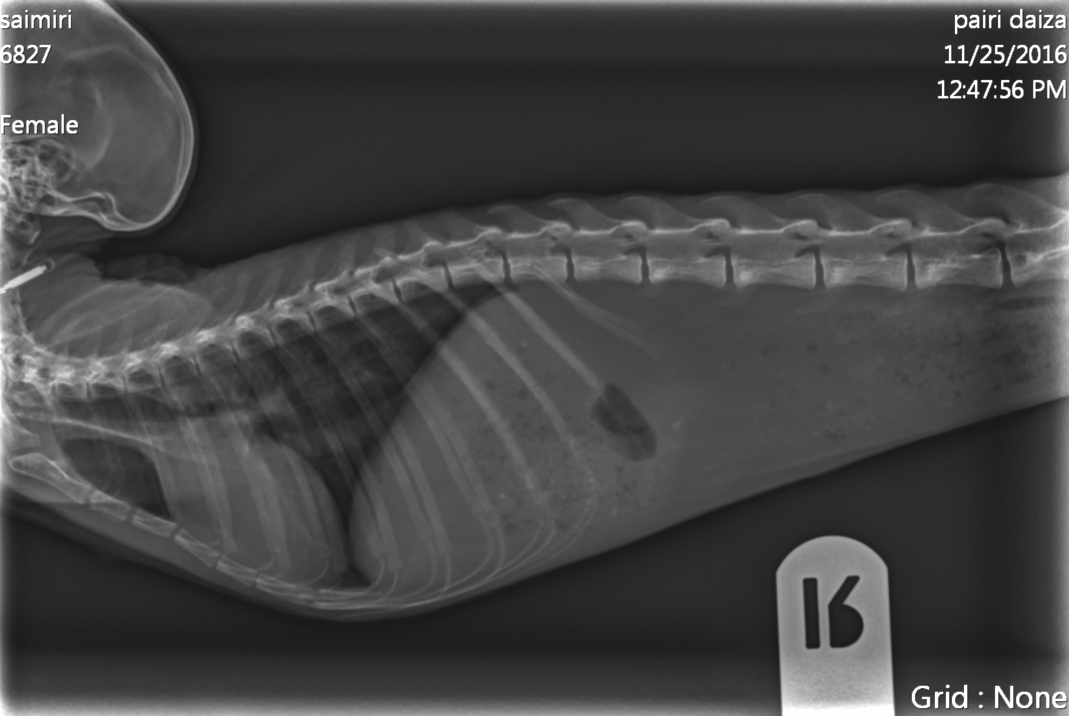

Supplement: S1 Fig — Size of the thoracic vertebrae, sternebrae, diameter of the aorta, caudal vena cava and angle of cardiac inclinication can be measured. (TIF) [file pone.0201646.s001.tif]

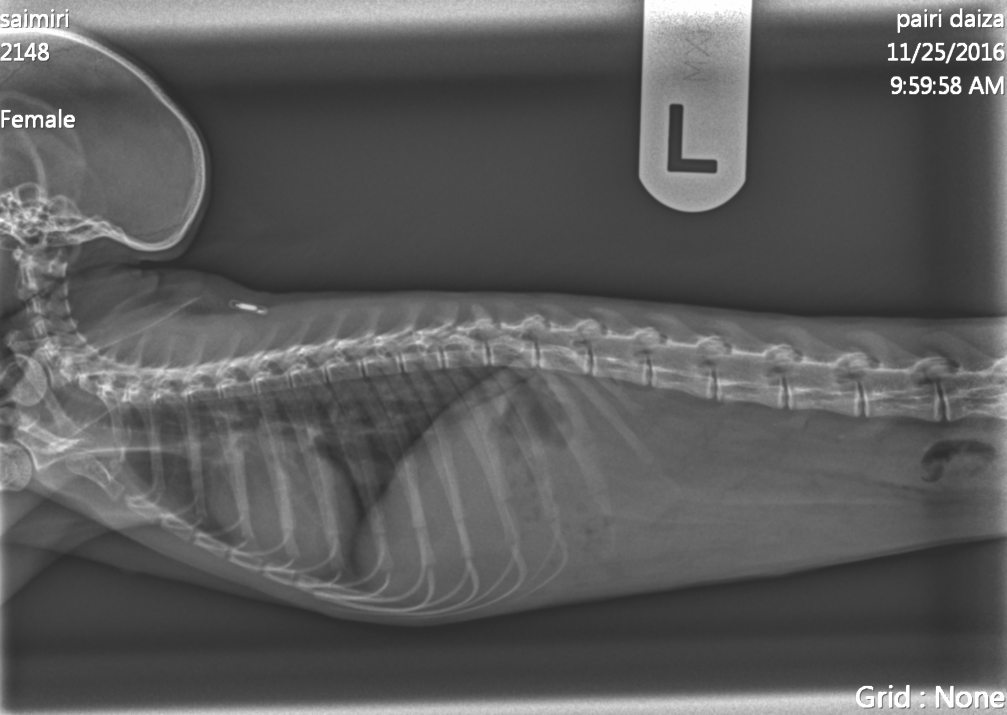

Supplement: S2 Fig — Tracheal diameter to thoracic inlet length ratio and tracheal inclination can be measured. (TIF) [file pone.0201646.s002.tif]

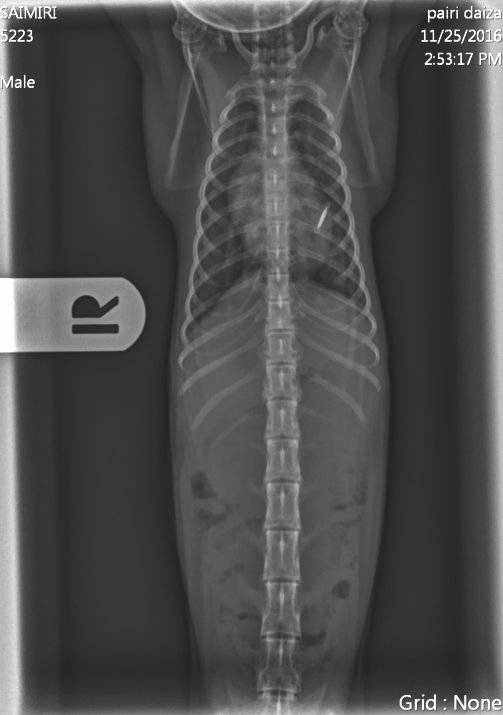

Supplement: S3 Fig — The cardio-thoracic, left and right costophrenic angles are measured. Note the presence of a clavicula. (TIF) [file pone.0201646.s003.tif]

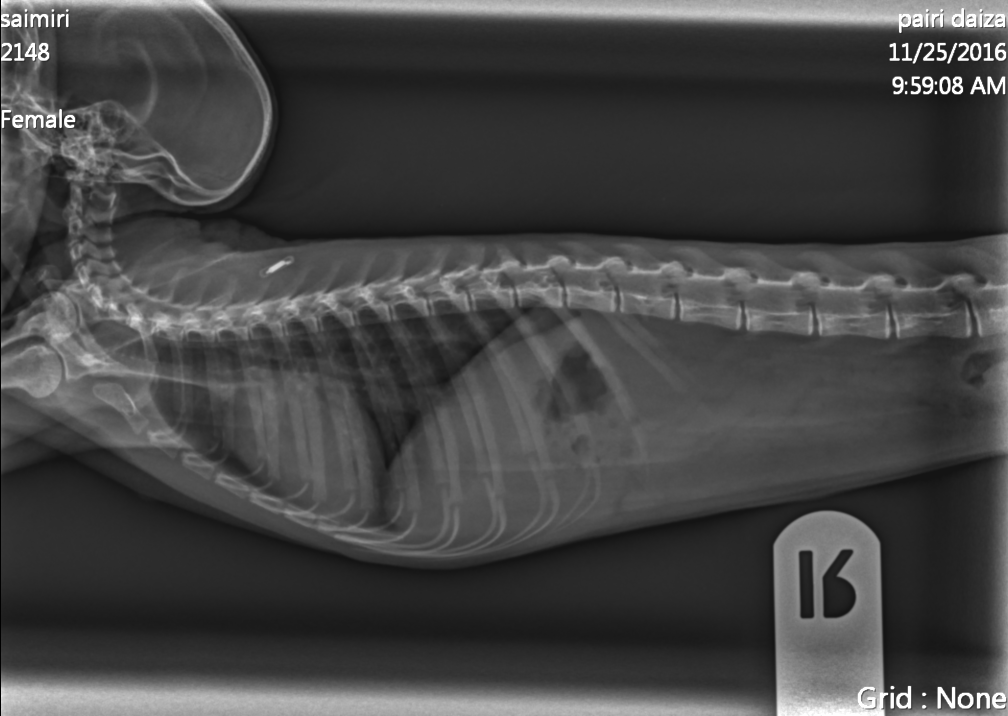

Supplement: S4 Fig — Measurement of the VHS (Vertebral Heart Score). (TIF) [file pone.0201646.s004.tif]
